# Moxibustion Exerts a Neuroprotective Effect through Antiferroptosis in Parkinson's Disease

**DOI:** 10.1155/2019/2735492

**Published:** 2019-07-31

**Authors:** Juan Lu, Xuelei Liu, Ye Tian, Hang Li, Zhenxing Ren, Shuang Liang, Guiyu Zhang, Caiping Zhao, Xinrong Li, Tingting Wang, Dongfeng Chen, Weihong Kuang, Meiling Zhu

**Affiliations:** ^1^Shenzhen Baoan Traditional Chinese Medicine Hospital, Guangzhou University of Chinese Medicine, Yuan 2 Road No. 25, Baoan District, Shenzhen 518133, China; ^2^Quanzhou Medical College, Fujian 362100, China; ^3^School of Basic Medical Science & Research Center of Basic Integrative Medicine, Guangzhou University of Chinese Medicine, Guangzhou 510006, China; ^4^Graduate School, Guangzhou University of Chinese Medicine, Guangzhou 510405, China

## Abstract

The objective of this study was to explore the neuroprotective effect of moxibustion on rats with Parkinson's disease (PD) and its mechanism. A Parkinson's disease model was established in rats using a two-point stereotactic 6-hydroxydopamine injection in the right substantia nigra (SN) and ventral tegmental area. The rats received moxibustion at the Baihui (GV20) and Sishencong (EX-HN1) acupoints for 20 minutes, six times a week, for 6 weeks. The right SN tissue was histologically and immunohistochemically examined. Differentially expressed genes (DEGs) were identified through RNA sequencing. In addition, the levels of tyrosine hydroxylase (TH), glutathione peroxidase 4 (GPX4), and ferritin heavy chain 1 (FTH1) in SN were measured. In comparison to the model group, the moxibustion group showed a significantly greater TH immunoreactivity and a higher behavioural score. In particular, moxibustion led to an increase in the number and morphological stability of SN neural cells. The functional pathway analysis showed that DEGs are closely related to the ferroptosis pathway. GPX4 and FTH1 in the SN were significantly overexpressed in the moxibustion-treated rats with PD. Moxibustion can effectively reduce the death of SN neurons, decrease the occurrence of ferroptosis, and increase the TH activity to protect the neurons in rats with PD. The protective mechanism may be associated with suppression of the ferroptosis.

## 1. Introduction

Parkinson's disease (PD) is the second most common age-related neurodegenerative movement disorder after Alzheimer's disease (AD) [1]. It is estimated that approximately 2% of the people older than 60 years of age have PD due to population aging [2]. PD is primarily characterized by degeneration of the dopaminergic neurons in the substantia nigra pars compacta (SNpc) [3], which results in movement disorders, including tremor, rigidity, gait difficulty, and bradykinesia [4]. Even though the pathogenesis of PD remains uncertain, it has been shown that iron accumulation plays a pivotal role in the development of PD, in the SNpc of postmortem brains of patients as well as in all PD animal models [5–7]. Ferroptosis is distinct from apoptosis, necroptosis, classic necrosis, and other reported forms of cell death by numerous criteria [7]. Ferritin heavy chain 1 (FTH1) is the target of iron death. As a major iron storage protein, FTH1 plays an important role in maintaining the iron balance in cells, and also plays an important regulatory role in a variety of signalling pathways [8]. Ferroptosis is an iron-dependent lipid peroxidation, which can be inhibited by the key ferroptosis regulator, glutathione peroxidase 4(GPX4), radical trapping antioxidants, and ferroptosis-specific inhibitors, such as ferrostatins and liproxstatins, as well as iron chelation [9]. Unfortunately, the current therapeutic options are limited [10]; pharmacotherapy fails to stop or even delay the dopaminergic neurodegeneration and its long-term use is associated with many adverse effects. All of the motor complications have been found to occur in 40% and 70% of the patients after 5- and 15-year levodopa treatment, respectively [11]. The patients gradually deteriorate to a higher rate of disability, which would bring an enormous economic burden and mental stress to the family and society [12]. The need for PD treatment strategies has led to the investigation of alternative therapies.

Moxibustion is an external therapeutic method of traditional Chinese medicine that exists for at least 3000 years. It has been used for treating PD and it has shown some positive results. It has been estimated that more than one-third of the patients with PD in the United States (40%) and the United Kingdom (38.7%) have already accepted at least one form of complementary and alternative medicine for PD, whereas 7%–10% of them have used acupuncture and moxibustion [13]. It was recently shown that moxibustion can postpone the progression of PD and decrease the symptom severity [14, 15]. Additionally, it has been shown to enhance the protective effect of dopamine neurons in patients with PD [16, 17]. Numerous studies have confirmed that moxibustion can improve the abnormal behaviour of mice with PD and reduce the loss of dopaminergic neurons in the SN. The acupoint selection is crucial during moxibustion. Baihui (GV20), as one of the points on the Governor Vessel, is situated at the midpoint of the line connecting the two ears. Since the head is the convergence of the Governor Vessel with all yang meridians, Baihui (GV20) can thereby regulate the yang qi of all yang meridians. In addition, the Governor Vessel which connects the brain can directly regulate the functions of the central nervous system. Sishencong (EX-HN1), as extra acupoints, are located 1 cun in front of, at the back of, and lateral to Baihui (GV20). Combined with Baihui (GV20), they can promote the functions of activating the brain and regulating the mind. In our study, Baihui (GV20) and Sishencong (EX-HN1) were selected as the main acupoints.

Moxibustion could regulate the content of superoxide dismutase and lipid peroxide in a rat model of PD [18]. Furthermore, moxibustion therapy was found to increase the antioxidative ability and the neuronal activity [19, 20]. Therefore, our study examined the effects of moxibustion on the ferroptosis and the expression of tyrosine hydroxylase (TH), GPX4, and FTH1 in a rat model of PD induced with 6-hydroxydopamine (6-OHDA).

## 2. Materials and Methods

### 2.1. Animals' Preparation

A total of 65 adult male Sprague-Dawley rats (12-week-old, beginning weight: 190–210 g) were used in the current study. All animals were obtained from Beijing Hua Fu kang Biotechnology Co. Ltd. (License number: SCXK (Beijing) 2014-0004; Beijing, China) and were raised in clean cabinets with free access to water and food. A controlled environment with a temperature of 20 ± 1°C, a humidity of 50%, and a 2-hour light-dark cycle was maintained throughout the study. This study's protocol was reviewed and approved by the Animal Care and Use Committee at the Peking University Shenzhen Graduate School (Shenzhen, China). All study conduct adhered to the World Health Organization's International Guiding Principles for Biomedical Research Involving Animals.

### 2.2. Grouping and Surgical Protocol

*Control Groups. *This study included two control groups of 12 rats each. Rats that were randomly placed into the normal control group (Normal) did not have any experimental interventions performed. Rats that were randomly placed into the sham control group (Sham) underwent an operation, but were administered 4 *μ*L of saline that contained 0.02% ascorbic acid instead of 6-OHDA. The injection procedures are described below.

*Parkinson's Disease Group. *The following procedures were performed to induce the onset of PD in this group of rats. After inducing anaesthesia with an intraperitoneal injection of chloral hydrate (400 mg/kg), rats were placed in the flat skull position on a cotton bed placed on a stereotaxic frame (Stoelting Co., Wood Dale, IL) that had been fitted with an incisor bar fixed 3.0 mm below the interaural line. Brain lesions were made by injecting 6-OHDA (16 *μ*g in 4 *μ*L 0.02% ascorbic acid saline solution) into two sites (AP: -4.9 mm, L: -1.9 mm, DV -7.5 mm and AP: -4.9 mm, L: -1.1 mm, DV: -8.0 mm relative to the bregma and dural surface) in the right SN and ventral tegmental area. Both injections were administered with a 5 *μ*L Hamilton microsyringe fitted at a rate of 0.5 *μ*L/min through 1-mm diameter burr holes drilled into the skull. After the injection, the needle was left in place for 10 minutes and then slowly withdrawn at a rate of 1 mm/min. Following the injections, a piece of gelatine sponge was placed into the burr holes to prevent bleeding and cerebral spinal fluid leakage. After the surgery, the rats were returned to their controlled environment cages. Unfortunately, 2 rats that had received 6-OHDA died following the injection. No deaths occurred in either the Normal group or the Sham group.

Rats with unilateral 6-OHDA lesions in the SN and ventral tegmental area were tested 4 weeks after the 6-OHDA injection for rotational asymmetry following intraperitoneal apomorphine (APO) administration (0.5 mg APO/kg in 0.9% saline). The recording began 10 minutes after the APO injection and the number of complete body turns (360°) in the direction ipsilateral to the lesion was counted. Rats making more than 7 nets full turns/min were classified as PD rats and were used in the next experimentation. In the end, no animals in the normal control group or the sham control group exhibited rotational behaviour following the APO administration. A total of 24 rats were classified as PD rats following the APO administration. The 24 rats reaching the preset definition of successfully induced hemi-Parkinsonism were randomly and evenly divided into the following two groups for treatment (n=12 each): model group (PD) and moxibustion group (Moxa).

### 2.3. Treatment and Techniques

The moxibustion was administered after the rotational behaviour test. The rats in the Normal and Sham groups, as well as the rats in the PD group received a normal feeding and did not undergo any therapeutic interventions. The rats in the Moxa group were treated with moxa stick at the Baihui (GV20) and Sishencong (EX-HN1) acupoints using the Han's Accupoint. A moxa is 7 mm in diameter and 35 mm long (Shenzhen Qianhai Ai Post Biotechnology Co. Ltd. Shenzhen, China). The moxa stick is inserted into the pedestal, which is 41-mm high, with a diameter of 30 mm. To reach the acupoints Baihui (GV20) and Sishencong (EX-HN1), an area of about 1.5 cm × 1.5 cm on the rats' head was shaved to expose the skin for moxibustion. The moxibustion treatment is a procedure of moxibustion of 20 minutes each time, six times a week for 6 weeks.

At the end of the treatment period of the Moxa group, the rotational behaviour test was performed on all rats of all four groups, the Normal, Sham, PD, and the Moxa group.

### 2.4. Assessment

*Histology. *After completion of the final behavioural testing, six of the 12 rats from each group were randomly selected to be sacrificed for histological assessment. Rats were anesthetized with 10% chloral hydrate (4 ml/kg) and fixed with aortal perfusion of 200 ml 0.9% normal saline (NS, to flush out the blood) followed by 300-400 ml of 4% paraformaldehyde solution in phosphate buffer (pH 7.4). After fixation, the brain tissue was gently removed, placed in a 4% paraformaldehyde solution, and embedded in paraffin. The paraffin blocks were then frozen and sectioned into 5-*μ*m thick coronal slices. Some paraffin-embedded SN sections were stained with haematoxylin and eosin (H&E) and imaged with microscopy.

*Nissl's Staining.* The brain sections were immersed into cresyl violet stain in 56°C temperature box impregnation for 10 min, and rinsed with deionized water. Subsequently, the sections were put into Nissl Differentiation for a few seconds to 2 min, (it was observed under the microscope until the background was nearly colourless). Finally, they were coverslipped with resinous mountant, and then observed using a light microscope.

*Immunohistochemistry.* After sectioning into 5-*μ*m thick coronal slices, the paraffin sections were dewaxed, and hydration was performed via successive baths in xylene 15 min and ethanol absolute, ethanol 95%, ethanol 90%, and ethanol 70%, 10 min each. The slides were immersed three times in PBS containing 0.1% Triton X-100 for 5 min. They were rinsed three times with 5% BSA for blocking. After blocking, they were incubated overnight at 4°C with the primary antibody against neuronal TH (1:800, Millipore, MA, USA). Sections were washed in PBS three times, and incubated with secondary antibodies (1:5000, EARTH, CA, USA) in a humidified chamber for 1 h at room temperature. Then, the tissue sections were incubated using the Liquid DAB substrate chromogen system (BOSTER, Wuhan, China). Slices were then dehydrated, clarified, and sealed in a routine manner. Neurons that were positive for TH stained brown and were easily photographed using light microscopy (x400 magnification). Three nonoverlapping fields in each SN section were used to count the number of TH immunoreactive (IR) positive cells in each section. A total of six rats in each study group were analyzed in this way. Additionally, the image pro plus 6 Analysis System (Media Cybernetics, MD, USA) was used to measure the mean optical density of each section to further quantify the TH-positive neurons in the SN. All sections were coded before the examination so that all examiners were masked to the rat treatment assignment.

*RNA Sequencing. *Total RNA was extracted from the SN using Trizol (Invitrogen, Carlsbad, CA, USA) following the manual instruction. Total RNA was qualified and quantified using a Nano Drop and Agilent 2100 bioanalyser (Thermo Fisher Scientific, MA, USA). Then, the first-strand cDNA was generated in a first-strand reaction system using PCR; the second-strand cDNA was generated as well. The cDNA fragments with adapters were amplified by PCR, and the products were purified by Ampure XP Beads. The library was validated on the Agilent Technologies 2100 bioanalyser for quality control. The double-stranded PCR products above were heat denatured and circularized by the splint oligo sequence. The single strand circle DNA (ssCir DNA) were formatted as the final library. The final library was amplified with phi29 (Thermo Fisher Scientific, MA, USA) to make a DNA nanoball (DNB), which had more than 300 copies of one molecular. The DNBs were loaded into the patterned nanoarray and pair-end 100 bases reads were generated on BGIseq500 platform (BGI-Shenzhen, China). RNA sequencing could be modelled as a random sampling process, in which each reading is sampled independently and uniformly from every possible nucleotide in the sample. To improve the accuracy of the differentially expressed gene (DEG) result, we defined a gene as a DEG when the reading number fold-change ≥2 and Q-value≤0.001.

*Western Blot Analysis. *After completion of the final behavioural testing, the remaining 12 rats in each group were sacrificed and the SN was quickly dissected on the ice. The tissue was homogenized in 100 *μ*l RIPA buffer (Beyotime, Shanghai, China) containing 1 mM protease inhibitor cocktail (Sigma, Aldrich, USA). After incubation on ice for 60 min, homogenates were centrifuged at 12500 rpm for 20 min at 4°C. The supernatants were transferred to fresh tubes and the protein concentrations were determined using a BCA protein assay kit (Beyotime, Shanghai, China). Protein lysates (50 *μ*g) were resolved on 6-12% SDS-PAGE gels, electrotransferred onto PVDF membranes (Millipore, Bedford, MA, USA). After blocking with 5% nonfat dry milk (milk dissolved into 0.1 M PBS with 0.1% Tween-20) for 1 h at room temperature, the membranes were incubated with rabbit antibody to TH (1:1000 dilution; Millipore); rabbit antibody to GPX4 (1:1000 dilution; Abcam); rabbit antibody to FTH1 (1:1000 dilution; CST); and rabbit antibody to GAPDH (1:1000 dilution; Santa Cruz) at 4°C overnight. Subsequently, the membranes were washed with PBST three times and incubated with HRP-labelled secondary antibodies (1:5000 dilution, EARTH) for 1 hour at room temperature. Immunoreactivity was detected using an Enhanced Chemiluminescent (ECL) kit (Millipore, USA) for 30 s. The signal was detected using a Fluorchem E system (ProteinSimple). The band intensity of the proteins was quantified using the Alpha View SA 3.4 (ProteinSimple).

*Real-Time Quantitative PCR.* Tissues were lysed, and total RNA was extracted by the three-step TRIzol method according to the RNA extraction kit (Invitrogen Life Technologies, Carlsbad, CA, USA). About 2 *μ*g of total RNA were reverse-transcribed in a 25-*μ*l reaction using a reverse transcription system (Solarbio Biological Technology Co., Ltd., Beijing, China.). Reverse transcription: 4 *μ*L of RNA, 0.5 *μ*L of an upstream primer, and 0.5 *μ*L of a downstream primer were added in a 25-*μ*L reverse transcription reaction system. Reverse transcription cDNA kit, Taq enzyme, and dNTP were purchased from Solarbio, Beijing, China. PCR amplification: 12.5 *μ*L of Takara Taq, 2.5 *μ*L of buffer, 2 *μ*L of dNTP, 0.5 *μ*L of an upstream primer, 0.5 *μ*L of a downstream primer, and 2 *μ*L of cDNA were added in the reaction system for amplification. The reaction conditions were as follows: 30 cycles of 95°C for 10 seconds, 58°C for 30 seconds, 72°C for 6 seconds. All specimens were stored at 4°C [21]. PCR product detection and data processing: PCR products were electrophoresed on 1.5% red gel containing ethidium bromide. Absorbance was monitored by a gel-scanning system (Bio-Rad, Hercules, CA, USA). The absorbance ratio of the target gene to *β*-actin was calculated. The forward and reverse primers of TH, GPX4, and FTH1 were synthesized by Sangon Biotech (Shanghai) Co., Ltd., China (Table 1).

### 2.5. Statistical Analyses

All data analyses were performed using SPSS statistical software (ver. 22.0, SPSS, Inc., Chicago, IL). Data are presented as mean ± standard deviation where applicable. Single factor analysis of variance was used, and the study groups were compared using one-way ANOVA, followed by LSD Student-Newman-Keuls test. Statistical significance was set to* p *< 0.05, whereas a highly statistical significance was set to* p *< 0.01 and Very significant difference was set to* p* <0.001.

## 3. Results

### 3.1. Effects of Moxibustion on the Rotational Behaviour in PD rats

**Figure 1:**
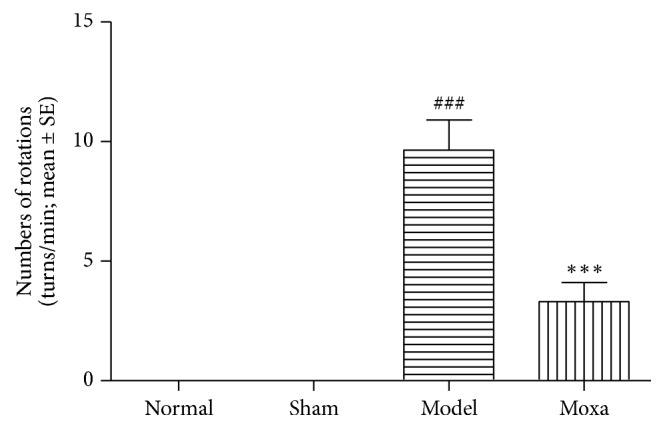
An apomorphine-induced rotation test to assess the motor behavioural changes in PD rats. Effects of the rotational behaviour in the following groups (*n* = 12 per group): normal control group, sham control group, model group, and moxibustion group. Data are presented as mean ± SE, one-way analysis of variance. Compared with the normal control group, ###*P*< 0.001; compared with the model group, *∗∗∗P*< 0.001.

No animals in the Normal or the Sham groups exhibited a rotational behaviour (0 turns/min) at any time point examined. However, the PD rats exhibited a rotational behaviour that was not significantly different after the treatment when the PD rats were examined together. However, the Moxa group showed a dramatic decrease in the number of APO-induced cycles after the treatment (*P *< 0.001) in comparison to the model group (*P* < 0.001; Figure 1).

### 3.2. Effects of Moxibustion on the substantia Nigra in PD Rats

**Figure 2:**
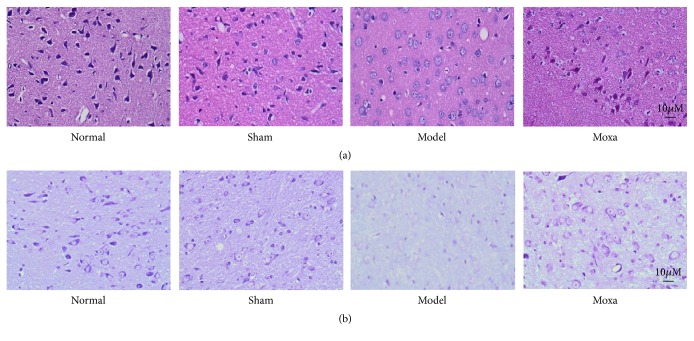
Effects of moxibustion on HE staining and Nissl's staining in the SN of 6-OHDA-induced PD model rats. (a) HE staining. (b) Nissl's staining. The shapes and sizes of neurons in the following groups (n = 6 per group): normal control group, sham control group, model group, and moxibustion group. Substantia nigra tissues were stained with H&E and Nissl's staining. Magnification: 400X.

Staining with H&E revealed no significant neuronal abnormalities in either the Normal or the Sham control groups, since many dopaminergic neurons were heavily stained and had normal shapes and sizes in these two groups. In contrast, there was an obvious decrease in the number of dopaminergic neurons in the PD group that was often accompanied by a disordered neuronal distribution, nuclear condensation, or swelling. Following the treatment, the SN dopaminergic neuron appearance significantly improved in the Moxa group, having normal morphology and smaller amounts of cellular degeneration (Figure 2(a)). Nissl's staining revealed clearly that the neurons are in normal cellular shape in both the Normal and the Sham groups. The cytoplasm was dyed deeply and evenly, with lots of huge tigroid Nissl bodies. In the PD group, Nissl bodies decreased remarkably in the 6-OHDA-lesioned SNpc, with obvious shrinking cells. The neurons and Nissl bodies in the Moxa group were denser than those in the PD group (Figure 2(b)).

### 3.3. Moxibustion Treatment Protects Dopamine Neurons in PD Rats

**Figure 3:**
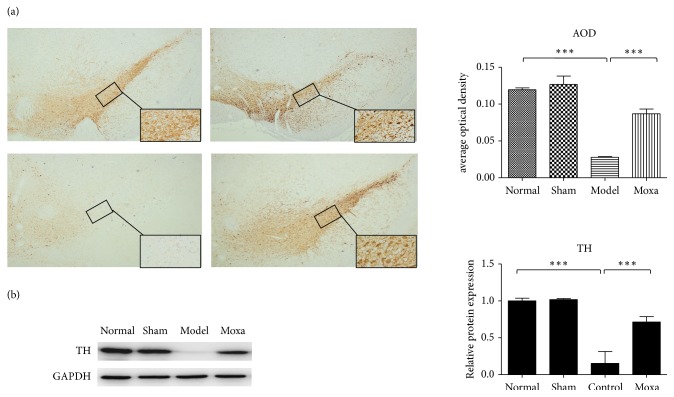
Moxibustion treatment improves the performance and protects the dopamine neurons in PD rats. (a) Effects of moxibustion on the TH-positive cells in SN tissue in the following groups (*n* = 6 per group, magnification: 40X and 400X): normal control group, sham control group, model group, and moxibustion group. Compared with the normal control group, **∗∗***P*< 0.01; compared with the model group, **∗***P*< 0.05. (b) TH-positive neurons counts in the rat SNpc in each group. Protein expression levels of TH were measured in the four groups. GAPDH was used as an internal control. Data were expressed as mean ± SD. **∗∗∗***P*< 0.001 vs. the normal control group; **∗∗***P*< 0.01 vs. the model group.

Immunohistochemistry staining with TH in SN tissue demonstrated that the Normal and Sham groups had significantly more TH-positive cells than the PD group (*P* < 0.01). A considerably lower number of TH-positive cells were observed in the SN tissue from PD rats. However, the animals in the Moxa group had higher numbers of TH-positive neurons than those in the PD group (P < 0.05, Figure 3(a)).

In this study, Western blot analysis showed that the expression of TH in the SN of rats was increased by the moxibustion treatment. Thus, moxibustion treatment can protects the dopaminergic neurons in PD rats (Figure 3(b)).

### 3.4. Signal Pathway Analysis

**Figure 4:**
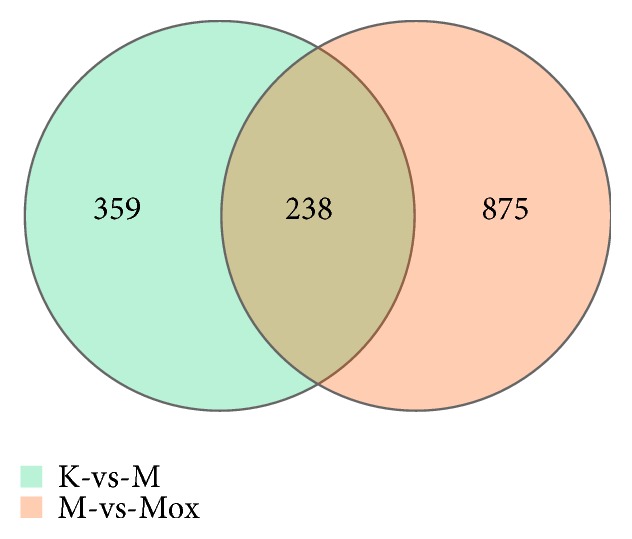
Venn diagram of differential expressed genes in the normal control group vs. the model group and the model group vs. the moxibustion group. Fold-change≥2 and Q-value≤0.001.

**Figure 5:**
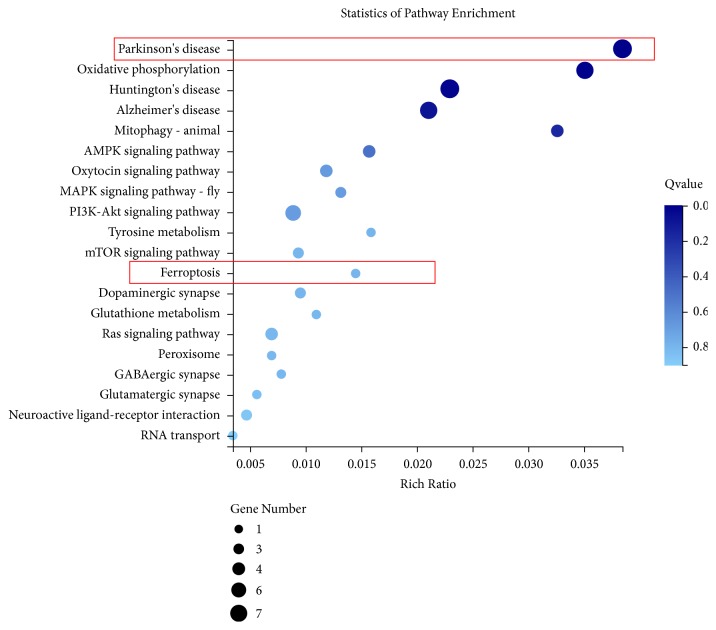
KEGG pathways of analysis. Fold-change≥2 and Q-value≤0.001.

Since functional pathway analysis showed that differential genes are closely related to the ferroptosis pathway, we conducted an in-depth study of the ferroptosis pathway to understand the action mechanism of moxibustion (Figure 4). For differentially expressed genes (DEGs) data from multiple comparison groups, we used Venn diagrams to show the presence of genes between different comparison groups. There were only 359 DEGs in the Normal and the PD group, 875 DEGs in the PD and the Moxa group, and 238 DEGs in all three groups (Fold-change≥2 and Q-value≤0.001, Figure 5).

### 3.5. Evidence of Ferroptosis in PD Rats after Moxibustion Treatment

**Figure 6:**
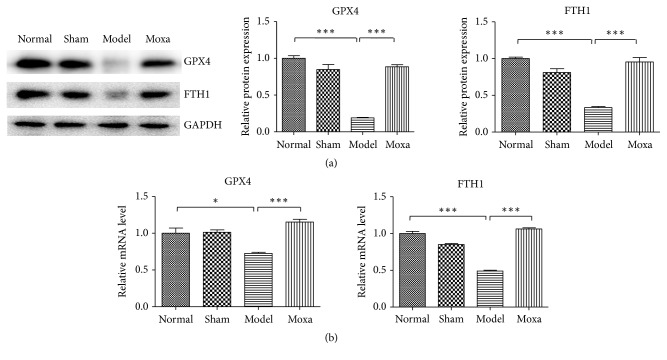
Evidence of ferroptosis in PD rats after moxibustion treatment. (a) The expression of GPX4 and FTH1 detected by Western blot analysis. The relative density of bands was calculated using GAPDH as a loading control. *∗∗∗P* < 0.001 (b) RT-PCR detection of GPX4 and FTH1 expression. The absorbance ratio of target gene to *β*-acting was calculated. *∗∗∗P*< 0.001, *∗P*< 0.05 vs. the normal control group; *∗∗∗P*< 0.001 vs. the model group.

Ferroptosis is distinct from classical apoptosis, autophagy, and necrosis in morphology. GPX4 and FTH1 were crucial during ferroptosis. In this study, the expression of GPX4, FTH1 protein, and mRNA in the SN of rats with PD was examined. The results showed that the expression of GPX4, FTH1 protein, and mRNA in the SN of rats was increased after the moxibustion treatment. The increased expression of GPX4 and FTH1 in SN of rats can inhibit the occurrence of ferroptosis and alleviate the damage of SN neurons in PD rats in the pathological process of PD. In summary, the moxibustion treatment protects the neuronal cells by inhibiting the activity of ferroptosis (Figure 6).

## 4. Discussion

In the present report, we established a Parkinson's disease (PD) model using a two-point stereotactic 6-OHDA injection in the right substantia nigra (SN) and ventral tegmental area. RNA-seq analysis revealed that ferroptosis correlates with the pathogenesis of PD. The moxibustion treatment improved the behavioural performance of the PD model rats and increased the expression of TH, GPX4, and FTH1 in the SN. Our results demonstrated that moxibustion can effectively reduce the death of substantia nigra neurons and the occurrence of ferroptosis. The TH activity is enhanced to protect the neurons in PD rats. The protective mechanism is related to the suppression of the ferroptosis.

Moxibustion is a traditional Chinese medicine therapy that consists of burning dried mugwort (moxa) on particular points (acupoint) on the body [21]. It is an external therapeutic method. Clinical meta-analysis has shown that moxibustion therapy has effect in the improvement of motor function and myotonia in patients with PD [14, 22]. Thus far, most of the relevant studies have focused on the effects and mechanisms of acupuncture or electro-acupuncture on PD [11, 23, 24]. It has been shown that acupuncture could obviously improve a series of symptoms of PD, including the motor dysfunction, depression, anxiety, and insomnia [25–27] by regulating the neurotropic factor-related gene and protein expression [28], antioxidation stress [29], anti-inflammation response [30], and neuron activity [31]. In comparison to acupuncture research, moxibustion can increase the parkin-mediated mitochondrial autophagy, promote the autophagic clearance of *α*-syn, and improve behavioural performance [21, 32]. Moxibustion has potential effect on cell repair and survival through the role of heat shock proteins [18]. Our study shows that moxibustion exerts a neuroprotective effect through antiferroptosis in PD. Acupoints play important roles in reliving the symptoms of PD. Yanglingquan (GB 34), Zusanli (ST36), Fengfu (GV16), Taichong (LR 3), Baihui (GV20), and Dazhui (GV14) acupoints have frequently been investigated for the effectiveness for treating PD [33]. In our study, Baihui (GV20) and Sishencong (EX-HN1) were selected as main points. Baihui (GV20) is situated at the top of the head, which is the convergence of the Governor Vessel with all yang meridians. The modern medical experiments confirmed that Baihui (GV20) can improve the cerebral blood circulation, enhance memory, and improve antidepressant activity, playing a role in brain protection [34–36]. Sishencong (EX-HN1) is widely applied in mental diseases, sleep disorders, cervical diseases, and cerebrovascular diseases [37, 38]. It can improve the cerebral microcirculation, influence sleep related factors, and regulate nerve excitability [39, 40]. Combined with Baihui (GV20), it can promote the functions of activating the brain and regulating the central nervous system. The research results have revealed that compared with the model group, moxibustion on Baihui and Sishencong points could significantly improve the praxiology of model rats. Furthermore, the abnormity of neurons in the SN of rats in the moxibustion group was alleviated, with an increased TH protein expression.

TH is very important in early onset of PD, because decreased TH activity and protein expression is a key link of DA deficiency and PD phenotypical expression [41–43]. Jellinger found that PD in the central nervous system are pathologically characterized by accumulation of *α*-syn Lewy body coatings, loss of TH-positive dopaminergic neurons in SN, and depletion of striatal dopamine [44]. Thenral* et al.* discovered that the TH mRNA content of patients with PD in the peripheral blood is lower than that in healthy people [45]. Our research results showed that the TH expression was significantly reduced in the PD model rats, which is consistent with the previously reported findings [46, 47]. Considering such rats' behaviours after modelling as significant rise in rotational behaviours and number of turns, retardation, muscle tremble and limb jitter, we could conclude that our model has hindered the TH expression in the SN of the rats, which further caused PD motor symptoms. Owing to the important role of TH in PD development, supplementing the TH content in the brain is one of the therapies. For example, Ugrumov proved that increasing the TH gene expression to make up for the declining TH content and activity is an effective way to treat PD mice in the presymptomatic stage [48].

Ferroptosis is an iron-dependent cell death pathway that involves depletion of intracellular reduced-glutathione (GSH) levels (the major antioxidant of neurons and natural ligand for iron in the ‘labile iron pool' (LIP)) and lipid peroxidation [49, 50]. The study found that ferroptosis plays a key role in the development of PD [51]. Iron imbalance, oxidative stress, and glutamic acid abnormality have important effects on ferroptosis and neurological diseases, accompanied by obvious changes in cell morphology, such as cell swelling, nuclear disruption, membrane blebbing, and distinctly altered mitochondrial morphology [52]. In our present studies, we found that ferroptosis is involved in the pathogenesis of PD based on RNA-seq sequencing technology, and FTH1 is the target of iron death. FTH1, as a major iron storage protein, plays an important role in maintaining the iron balance in cells and also plays an important regulatory role in a variety of signalling pathways [8]. Excess iron is stored in ferritin. When the expression of ferritin FTH1 decreases, iron storage reduces. Excess iron may lead to ferroptosis [53]. Failure of the lipid repair enzyme GPX4 causes accumulation of reactive oxygen species (ROS) on the membrane lipids [9, 54]. In our experiments, moxibustion increased the content of GPX4 and FTH1 in PD rats, inhibited the progression of iron death, and increased the number of TH-positive cells in the SN.

## 5. Conclusions

In summary, moxibustion can effectively reduce the death of substantia nigra neurons and the occurrence of ferroptosis and increase the TH activity to protect neurons in PD rats. The protective mechanism is related to the suppression of ferroptosis. Moxibustion therapy can increase the antioxidative capacity and regulate neuronal activity, both of which are curative on PD. This complementary effect of moxibustion therapy will be a focus of our future research.

## Figures and Tables

**Table 1:** Primer sequences employed for reverse-transcription PCR and anticipated product size.

Gene	Primer	Sequence (5'-3')	Product size(bp)
TH	Forward	ATTGCCTTCCAGTACAAGCAC	97
	Reverse	CCTTCAGCGTGACATATACCTCC	
GPX4	Forward	ATAAGAACGGCTGCGTGGTGAAG	82
	Reverse	TAGAGATAGCACGGCAGGTCCTTC	
FTH1	Forward	TTCAGGGCCACATCATCCCG	129
	Reverse	GCAAGTGCGCCAGAACTACC	
*β*-acting	Forward	CTCAGGAGAGGAGCCATTTATT	115
	Reverse	CCCGATCAGAGTGAAGCTATT	

## Data Availability

The data used to support the findings of this study are available from the corresponding author upon request.
